# Basal knowledge in the field of pediatric nephrology and its enhancement following specific training of ChatGPT-4 “omni” and Gemini 1.5 Flash

**DOI:** 10.1007/s00467-024-06486-3

**Published:** 2024-08-16

**Authors:** Gianluca Mondillo, Vittoria Frattolillo, Simone Colosimo, Alessandra Perrotta, Anna Di Sessa, Stefano Guarino, Emanuele Miraglia del Giudice, Pierluigi Marzuillo

**Affiliations:** Department of Woman, Child and of General and Specialized Surgery, Università Degli Studi Della Campania “Luigi Vanvitelli”, Via Luigi De Crecchio 2, 80138 Naples, Italy

**Keywords:** Artificial intelligence, Pediatric nephrology, Gemini 1.5, ChatGPT-4o

## Abstract

**Background:**

We aimed to evaluate the baseline performance and improvement of ChatGPT-4 “omni” (ChatGPT-4o) and Gemini 1.5 Flash (Gemini 1.5) in answering multiple-choice questions related to pediatric nephrology after specific training.

**Methods:**

Using questions from the “Educational Review” articles published by Pediatric Nephrology between January 2014 and April 2024, the models were tested both before and after specific training with Portable Data Format (PDF) and text (TXT) file formats of the Educational Review articles removing the last page containing the correct answers using a Python script. The number of correct answers was recorded.

**Results:**

Before training, ChatGPT-4o correctly answered 75.2% of the 1395 questions, outperforming Gemini 1.5, which answered 64.9% correctly (*p* < 0.001). After training with PDF files, ChatGPT-4o’s accuracy increased to 77.8%, while Gemini 1.5 improved significantly to 84.7% (*p* < 0.001). Training with TXT files showed similar results, with ChatGPT-4o maintaining 77.8% accuracy and Gemini 1.5 further improving to 87.6% (*p* < 0.001).

**Conclusions:**

The study highlights that while ChatGPT-4o has strong baseline performance, specific training does not significantly enhance its accuracy. Conversely, Gemini 1.5, despite its lower initial performance, shows substantial improvement with training, particularly with TXT files. These findings suggest Gemini 1.5’s superior ability to store and retrieve information, making it potentially more effective in clinical applications, albeit with a dependency on additional data for optimal performance.

**Graphical Abstract:**

**Figure:**
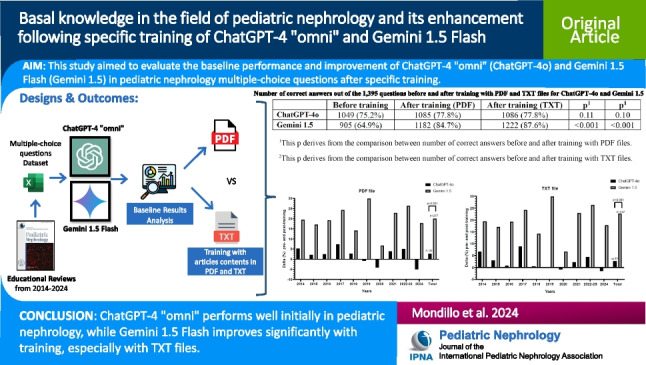
A higher resolution version of the Graphical abstract is available as Supplementary information

**Supplementary Information:**

The online version contains supplementary material available at 10.1007/s00467-024-06486-3.

## Introduction

Large language models (LLMs) have transformed the field of artificial intelligence and natural language processing [1]. LLMs are algorithms based on deep neural networks designed to process and generate natural language text [2, 3]. They are trained on massive datasets that include books, scientific articles, websites, and other text sources, allowing them to acquire a wide range of knowledge [4].

Among the most advanced LLMs currently available are ChatGPT-4 “omni” (ChatGPT4o) [5] and Gemini 1.5 Flash (Gemini 1.5) [6]. Each has distinctive technical features and specific applications, including the medical-scientific field. ChatGPT-4o integrates multimodal capabilities that include text, images, and audio, making it much faster and more efficient than its predecessors. It is accessible, with interaction limitations, to even the free-tier users of ChatGPT. Its applications range from real-time translation to image analysis and natural voice conversation, making it a versatile tool for numerous sectors, including medicine.

Gemini 1.5 has been optimized for speed and accuracy. This model stands out for its ability to handle complex queries and provide detailed and accurate responses in various contexts, including medical ones [7].

LLMs like ChatGPT-4o and Gemini 1.5 have shown remarkable potential in the medical field, being able to analyze clinical data, support diagnosis, provide therapeutic advice, and assist in the continuous training of healthcare professionals [8].

To the best of our knowledge, no studies have investigated the feasibility of these LLMs in the field of pediatric nephrology. We hypothesized that the LLMs could find some applications, in the future, in the support of Pediatric Nephrologists. Starting from this hypothesis, we aimed to conduct a comparative evaluation of the ChatGPT-4o and Gemini 1.5 models on multiple-choice questions in pediatric nephrology, both before and after specific training, to gain valuable insights into their capabilities and limitations in this specialized context.

## Methods

To evaluate the performance of ChatGPT-4o and Gemini 1.5 in answering pediatric nephrology multiple-choice questions, we adopted a rigorous and structured approach. The multiple-choice questions were collected from the “Educational Review” articles published by the journal Pediatric Nephrology between January 2014 and April 2024 [9]. These articles contain multiple-choice questions at the end of the texts, with the correct answers provided after the bibliography section.

We chose questions up to 2014 for benchmarking to avoid biases from changes in medical knowledge over time. Older questions might have answers considered correct at the time but are now deemed incorrect. This approach ensures a fair assessment of the models’ capabilities.

We tested the models with both Portable Data Format (PDF) and text (TXT) file formats to understand the impact of extraneous metadata and formatting complexities on the performance of LLMs. PDFs preserve visual integrity but contain complex layouts, making structured data extraction challenging. Issues like non-linear text storage and variable font encoding further complicate this [10].

In contrast, TXT files are simpler and lack these formatting complexities, making them easier for LLMs to process. Research indicates LLM performance is better with simpler, less noisy data formats, allowing for more accurate information retrieval and processing [11]. This hypothesis motivated our decision to compare the models’ performance across these two formats.

The questions, along with the multiple-choice answers, were organized into several Excel files, divided by year, and then presented to the ChatGPT-4o and Gemini 1.5 models. We have made the question dataset available on HuggingFace [12], while the codes used are available on GitHub [13].

The prompt used to present the questions to the models was as follows: “I will provide you with multiple-choice questions. You need to read and answer the questions with the letter of the answer you think is correct. Some questions may have more than one correct answer. Print two columns: the first with the question numbers, the second with the corresponding answers. Clear?” The answers provided by the models were then compared with those indicated as correct in the articles, assigning a value of 1 to the correct answers and 0 to the wrong ones. For questions with multiple correct answers, any responses that were only partially correct according to the solution key were considered wrong. We calculated the percentage of correct answers for each year and for the entire ten-year period. Due to the small number of questions in the 2022–2023 biennium, we decided to combine them and treat them as a single year.

Subsequently, we integrated the reference articles, removing the last page containing the correct answers using a Python script. The modified articles were combined into PDF files named “[year] FUSED.pdf” and presented to the models with the prompt: “Read and memorize the content of this file. I will ask you multiple-choice questions which you will need to answer based on the knowledge contained in this file.” The questions were then re-presented and analyzed using the same methodology described earlier.

To further evaluate the models’ effectiveness, the articles were also converted into TXT using another Python script we coded. Again, the models were tested with the multiple-choice questions using the same methodology.

To avoid interference between different interactions, we created a new chat session for each interaction with the models, minimizing learning from previous interactions. Moreover, during the experiments, no correct answers were provided to the models. For this reason, the models would not have had the opportunity to learn anything concrete from the interactions, as they did not receive corrective feedback.

The results obtained before and after presenting the PDF and TXT files were compared to evaluate the impact of the models’ memorization of the articles on the correct response rates.

### Statistical analysis

*P* values < 0.05 were considered statistically significant. Qualitative variables were compared by using the chi-square test. The chi-square test was conducted using the Stat-Graph XVII software for Windows, while the confusion matrix analysis was performed using Python (version 3.12.3), the libraries pandas (version 2.0.3) were used for data manipulation, NumPy (version 1.24.3) for array operations, scikit-learn (version 1.3.0) for creating the confusion matrix, and matplotlib (version 3.7.2) along with seaborn (version 0.12.2) for visualizing the matrix.

## Results

During the analysis period, we extracted 1395 questions from Educational Review articles published in Pediatric Nephrology.

Before specific training, GPT-4o correctly responded to 1049 out of 1395 questions (75.2%), while Gemini 1.5 correctly answered 905 questions (64.9%) (*p* < 0.001).

After training, which involved administering a PDF file containing all selected Educational Review articles, ChatGPT-4o correctly answered 1085 questions (77.8%), while Gemini 1.5 correctly responded to 1182 questions (84.7%) (*p* < 0.001).

Following training with TXT files, ChatGPT-4o correctly answered 1086 questions (77.8%), whereas Gemini 1.5 correctly responded to 1222 questions (87.6%) (*p* < 0.001).

**Fig. 1:**
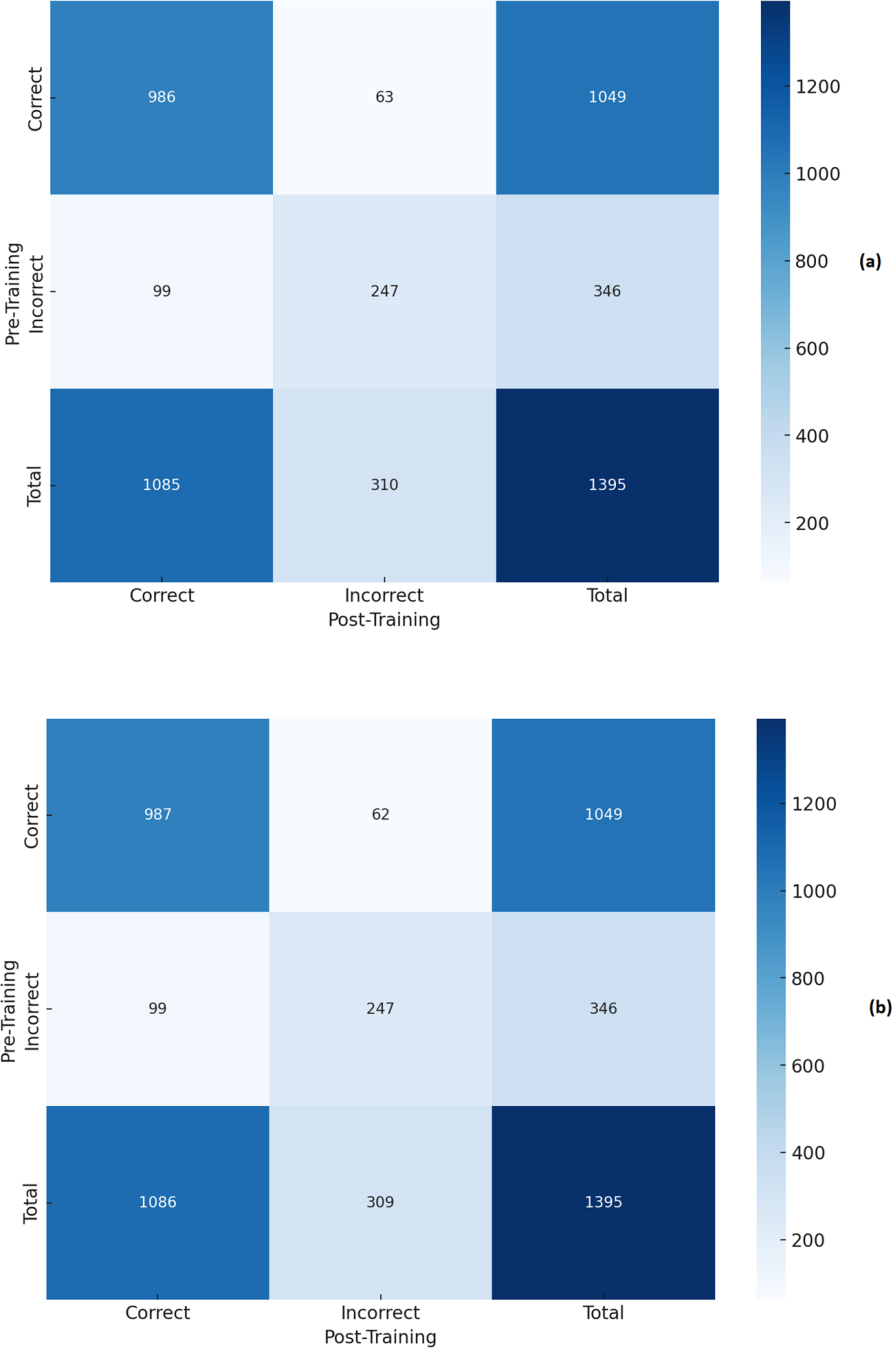
Confusion matrix analysis of the correct and incorrect answers, both before and after training with PDF (**a**) and TXT (**b**) files for ChatGPT-4.0

**Fig. 2:**
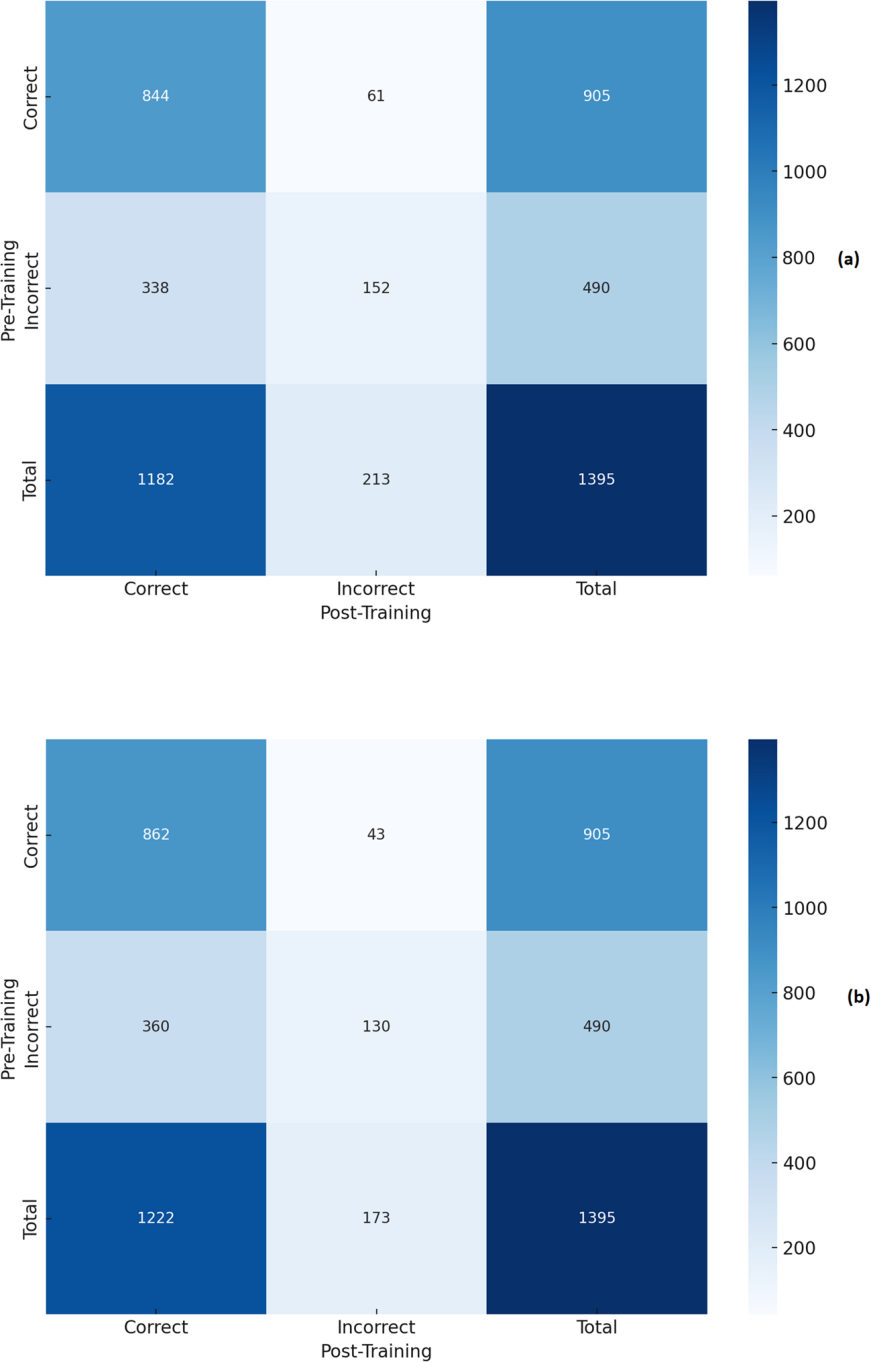
Confusion matrix analysis of the correct and incorrect answers, both before and after training with PDF (**a**) and TXT (**b**) files for Gemini 1.5

The confusion matrix analysis of the correct and incorrect answers, both before and after training with PDF and TXT files for ChatGPT-4o and Gemini 1.5, are shown in Fig. 1 and Fig. 2, respectively.

The incorrect answers before training alongside the answers given after training with PDF and with TXT are shown respectively in Supplementary Table 1 and in Supplementary Table 2 for ChatGPT-4o and in Supplementary Table 3 and Supplementary Table 4 for Gemini 1.5. On the other hand, the questions with correct answers before training and incorrect answers after training with PDF and with TXT are shown respectively in Supplementary Tables 5 and 6 for ChatGPT-4o and Supplementary Tables 7 and 8 for Gemini 1.5.

The percentage of correctly answered questions did not significantly improve after training for ChatGPT-4o, while it was significant for Gemini 1.5 (Table 1). Moreover, the improvement of the percentage of correct answers comparing training with PDF files vs. TXT files was not significant for both the LLMs (*p* = 0.96 for ChatGPT-4o and 0.09 for Gemini 1.5).

**Table 1:** Number of correct answers out of the 1,395 questions before and after training with PDF and TXT files for ChatGPT-4o and Gemini 1.5

	Before training	After training (PDF)	After training (TXT)	*p*^1^	*p*^2^
ChatGPT-4o	1049 (75.2%)	1085 (77.8%)	1086 (77.8%)	0.11	0.10
Gemini 1.5	905 (64.9%)	1182 (84.7%)	1222 (87.6%)	< 0.001	< 0.001

^1^This *p* derives from the comparison between the number of correct answers before and after training with PDF files

^2^This *p* derives from the comparison between the number of correct answers before and after training with TXT files

**Fig. 3:**
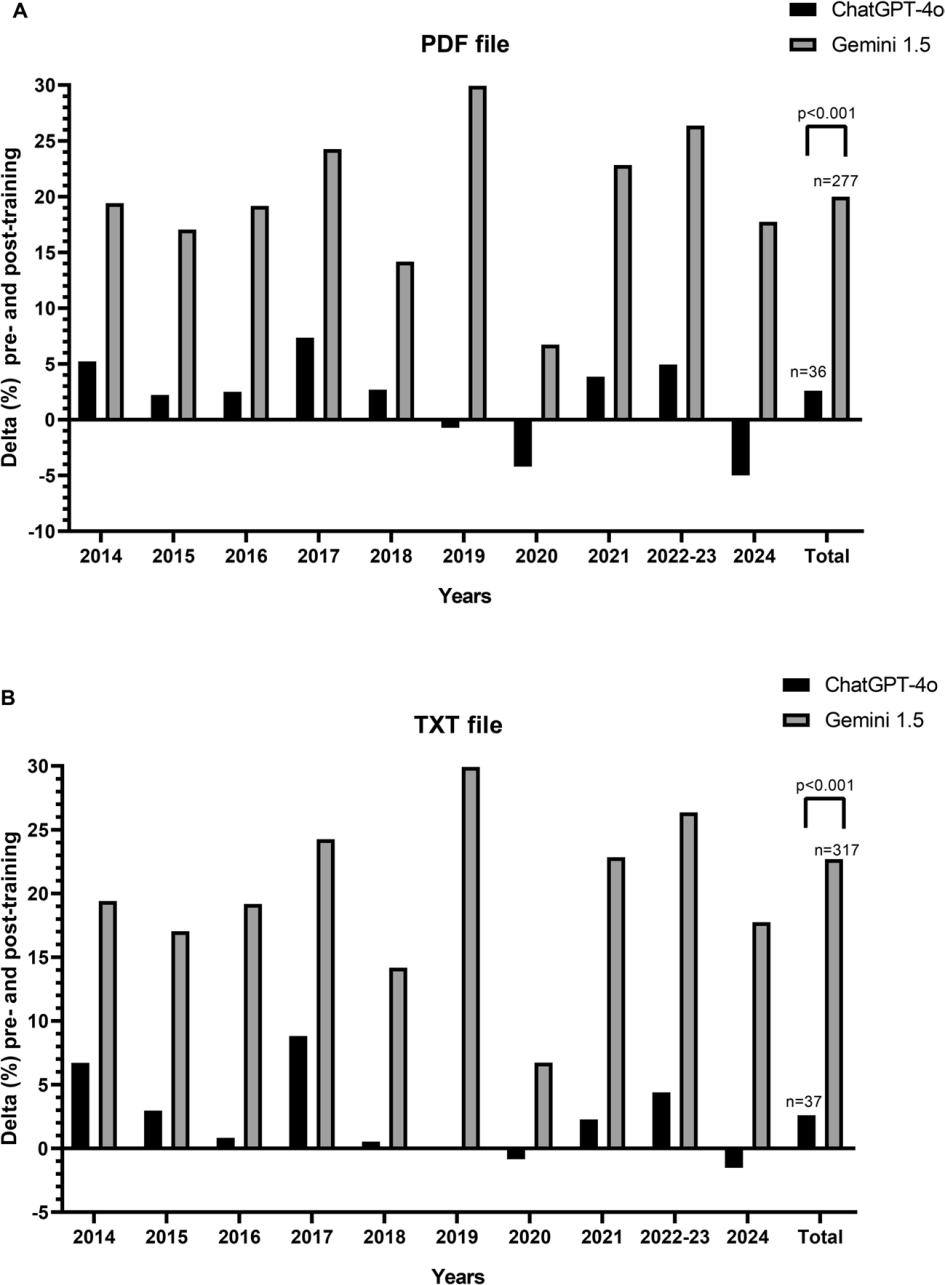
Pre- and post-training delta (%) for both ChatGPT-4o and Gemini 1.5, using a PDF file (Panel **A**) and a TXT file (Panel **B**) for training

The pre- and post-training delta for both ChatGPT-4o and Gemini 1.5 is shown in Fig. 3. For both the PDF and TXT file training, the sum of the delta was significantly higher for Gemini 1.5 compared to ChatGPT-4o (Fig. 3A and B).

## Discussion

At the time of writing, this represents the first study testing OpenAI (ChatGPT-4o) and Google (Gemini 1.5) new models in the medical field in general, and specifically in pediatric nephrology.

Previously, only one study on LLMs focused on the nephrology of adults, showing that ChatGPT-4 (the previous version) correctly answered 73.3% of the questions from the Nephrology Self-Assessment Program [15], significantly outperforming open-source models that scored between 17.1 and 30.6%.

Our data indicate that ChatGPT-4o, whose knowledge extends up to October 2023 (compared to December 2022 for ChatGPT-4) [16], presented an impressive performance without training. The percentage of correct answers was significantly higher (75.2% vs. 64.9%; *p* < 0.001) compared to Gemini 1.5.

Despite its poor initial performance, Gemini 1.5 showed significant improvement after the training (Table 1), even surpassing ChatGPT-4o (Fig. 1). On the other hand, ChatGPT-4o did not exhibit significant improvement after training (Table 1).

Training with PDF and TXT files resulted in similar performance for both LLMs, although we observed an interesting trend where training with TXT files led to greater improvement compared to PDF files for Gemini 1.5.

Interestingly, both ChatGPT-4o and Gemini 1.5 showed a decline in post-training improvement in the year 2020 (Fig. 3). We hypothesize that the decline in post-training improvement observed in 2020 can be attributed to various factors, including the COVID-19 pandemic and the quality of data from that year. The LLMs, in fact, in 2020 might have been inundated with specific COVID-19 data, limiting the variety of information on other topics and consequently reducing the effectiveness of learning on more general subjects. Artificial intelligences, particularly complex structures like LLMs, however, are “black box” models [17]. This means that we cannot know exactly what happens inside them, making it challenging to precisely identify the causes of certain behaviors [18].

The results of studies on the use of LLMs in the medical field reveal both the potential and challenges of integrating this language model into healthcare practices [19]. Data, for example, indicates that ChatGPT 3.5 has a good level of competence, although lower than that of expert physicians, suggesting the utility as an educational and diagnostic support tool [20]. Moreover, as found in our paper, only a 2.5% increase in accuracy after specific training was observed.

On the other hand, Med-Gemini, a specialized version of Gemini, has achieved state-of-the-art performance on various benchmarks with an accuracy of 91.1%, surpassing previous models like Med-PaLM 2 by 4.6% [21, 22].

In conclusion, our study indicates that ChatGPT-4o correctly answers 75.2% of the questions in the field of pediatric nephrology, and specific training does not improve its performance. On the other hand, Gemini 1.5 had a poorer performance compared to ChatGPT-4o without training but showed an impressive improvement after specific training. These findings suggest that Gemini 1.5 has a superior ability to store and retrieve information, making it potentially more effective in practical and clinical applications. However, the model still requires user data, limiting its standalone clinical use. ChatGPT-4o, on the other hand, exhibits less dependence on additional literature, demonstrating good baseline knowledge in specialized medical fields like pediatric nephrology.

## Supplementary Information

Below is the link to the electronic supplementary material.

Graphical abstract (PPTX 254 KB)

Supplementary file1 (XLSX 64.6 KB)

Supplementary file2 (XLSX 64.5 KB)

Supplementary file3 (XLSX 87.6 KB)

Supplementary file4 (XLSX 87.5 KB)

Supplementary file5 (XLSX 19.4 KB)

Supplementary file6 (XLSX 19.4 KB)

Supplementary file7 (XLSX 20.8 KB)

Supplementary file8 (XLSX 17.5 KB)

## Data Availability

The data (excel file) supporting the findings of this study are openly available in https://huggingface.co/datasets/GianlucaMondillo/PedNephrologyMCQ_Vanvitelli (accessed June 10, 2024) and https://github.com/GianlucaMondillo/PedNephrologyMCQ_Vanvitelli (accessed June 10, 2024).
