# Supplementary material for: Basal knowledge in the field of pediatric nephrology and its enhancement following specific training of ChatGPT-4 “omni” and Gemini 1.5 Flash
Source: Pediatr Nephrol. 2024 Aug 16;40(1):151–7. doi: 10.1007/s00467-024-06486-3 (PMC11584465; doi:10.1007/s00467-024-06486-3)
Supplement: Supplementary file 1 — Graphical abstract (PPTX 254 KB) [file 467_2024_6486_MOESM1_ESM.pptx]

## Slide 1
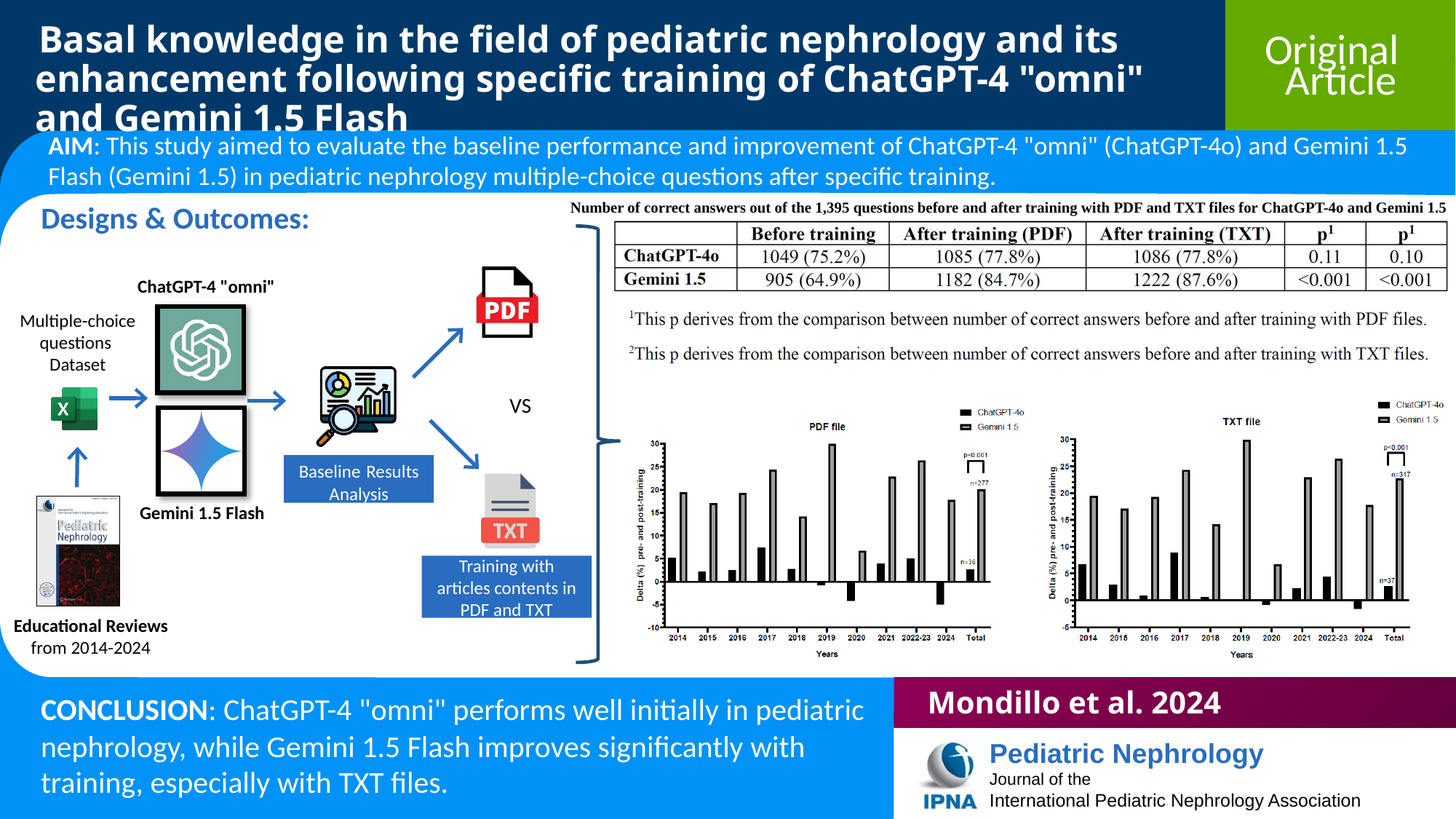

Basal knowledge in the field of pediatric nephrology and its enhancement following specific training of ChatGPT-4 "omni" and Gemini 1.5 Flash
AIM: This study aimed to evaluate the baseline performance and improvement of ChatGPT-4 "omni" (ChatGPT-4o) and Gemini 1.5 Flash (Gemini 1.5) in pediatric nephrology multiple-choice questions after specific training.
Number of correct answers out of the 1,395 questions before and after training with PDF and TXT files for ChatGPT-4o and Gemini 1.5
Designs & Outcomes:
ChatGPT-4 "omni"
Multiple-choice questions
Dataset
VS
Baseline Results Analysis
Gemini 1.5 Flash
Training with articles contents in PDF and TXT
Educational Reviews
from 2014-2024
Mondillo et al. 2024
CONCLUSION: ChatGPT-4 "omni" performs well initially in pediatric nephrology, while Gemini 1.5 Flash improves significantly with training, especially with TXT files.
